# Commencement of the Philadelphia College of Dental Surgery

**Published:** 1853-04

**Authors:** 


*Commencement of the Philadelphia College of Dental Surgery.*—The first annual commencement of this Institution was held on the 28th of February, 1853, at the Sansom street Hall, Philadelphia. The degree was conferred by the dean of the faculty, Professor E. Townsend, on the following gentlemen, S. T. Brown, of Penn.; G. W. Emerson, D. C.; J. S. Gilliams, Philadelphia; H. Garrett, Del.; R. A. Miller, Penn.; A. B. Williams, D. C.

The honorary degree was at the same time conferred on Wm. Bradly, M. D.; S. T. Beal, M. D.; S. Dillingham, J. F. Flagg, M. D.; W. W. Fouche, Jacob Gilliams, M. D.; J. M. Harris, M. D.; J. H. McQuillen, M. D.; S. L. Mintzer, Daniel Neall, F. Reinstein, Edward Townsend, Charles Townsend, Jun.; D. B. Whipple, M. D.; C. C. Williams, S. S. White, of Philadelphia; T. W. Evans, of Paris; J. F. Flagg, M. D., of Boston; J. Flemming, M. D., of Harrisburg, Penn.; O. R. Post, of Brattleboro, Vt.; and W. B. Webster, of Richmond, Indiana.

This part of the ceremony having been gone through with, Professor J. D, White delivered a very appropriate and able valedictory address.

We had expected to have been present on the occasion of this the first commencement of this Institution—having went to Philadelphia with Professor Bond for that purpose, but we did not arrive there until after the ceremonies were concluded. We have subsequently, however, had the pleasure of reading the valedictory, which has been published in the Dental News Letter. Again, we wish the Philadelphia College success. The expectations of the friends of the school thus far, have, we are happy to learn, been more than realized.

